# Genetic variants underlying risk of endometriosis: insights from meta-analysis of eight genome-wide association and replication datasets

**DOI:** 10.1093/humupd/dmu015

**Published:** 2014-03-27

**Authors:** Nilufer Rahmioglu, Dale R. Nyholt, Andrew P. Morris, Stacey A. Missmer, Grant W. Montgomery, Krina T. Zondervan

**Affiliations:** 1 Wellcome Trust Center for Human Genetics, University of Oxford, Roosevelt Drive, Oxford OX3 7BN, UK; 2 Neurogenetics, QIMR Berghofer Medical Research Institute, Brisbane QLD 4029, Australia; 3 Department of Biostatistics, University of Liverpool, Duncan Building, Daulby Street, Liverpool L69 3GA, UK; 4 Department of Obstetrics, Gynecology and Reproductive Biology, Brigham and Women's Hospital and Harvard Medical School, 75 Francis Street, Boston, MA 02115, USA; 5 Molecular Epidemiology, QIMR Berghofer Medical Research Institute, Brisbane QLD 4029, Australia; 6 Nuffield Department of Obstetrics and Gynaecology, University of Oxford, John Radcliffe Hospital, Oxford OX3 9DU, UK

**Keywords:** endometriosis, genetics, GWAS, sub-phenotypes, heterogeneity

## Abstract

**BACKGROUND:**

Endometriosis is a heritable common gynaecological condition influenced by multiple genetic and environmental factors. Genome-wide association studies (GWASs) have proved successful in identifying common genetic variants of moderate effects for various complex diseases. To date, eight GWAS and replication studies from multiple populations have been published on endometriosis. In this review, we investigate the consistency and heterogeneity of the results across all the studies and their implications for an improved understanding of the aetiology of the condition.

**METHODS:**

Meta-analyses were conducted on four GWASs and four replication studies including a total of 11 506 cases and 32 678 controls, and on the subset of studies that investigated associations for revised American Fertility Society (rAFS) Stage III/IV including 2859 cases. The datasets included 9039 cases and 27 343 controls of European (Australia, Belgium, Italy, UK, USA) and 2467 cases and 5335 controls of Japanese ancestry. Fixed and Han and Elkin random-effects models, and heterogeneity statistics (Cochran's *Q* test), were used to investigate the evidence of the nine reported genome-wide significant loci across datasets and populations.

**RESULTS:**

Meta-analysis showed that seven out of nine loci had consistent directions of effect across studies and populations, and six out of nine remained genome-wide significant (*P* < 5 × 10^−8^), including rs12700667 on 7p15.2 (*P* = 1.6 × 10^−9^), rs7521902 near *WNT4* (*P* = 1.8 × 10^−15^), rs10859871 near *VEZT* (*P* = 4.7 × 10^−15^), rs1537377 near *CDKN2B-AS1* (*P* = 1.5 × 10^−8^), rs7739264 near *ID4* (*P* = 6.2 × 10^−10^) and rs13394619 in *GREB1* (*P* = 4.5 × 10^−8^). In addition to the six loci, two showed borderline genome-wide significant associations with Stage III/IV endometriosis, including rs1250248 in *FN1* (*P* = 8 × 10^−8^) and rs4141819 on 2p14 (*P* = 9.2 × 10^−8^). Two independent inter-genic loci, rs4141819 and rs6734792 on chromosome 2, showed significant evidence of heterogeneity across datasets (*P* < 0.005). Eight of the nine loci had stronger effect sizes among Stage III/IV cases, implying that they are likely to be implicated in the development of moderate to severe, or ovarian, disease. While three out of nine loci were inter-genic, the remaining were in or near genes with known functions of biological relevance to endometriosis, varying from roles in developmental pathways to cellular growth/carcinogenesis.

**CONCLUSIONS:**

Our meta-analysis shows remarkable consistency in endometriosis GWAS results across studies, with little evidence of population-based heterogeneity. They also show that the phenotypic classifications used in GWAS to date have been limited. Stronger associations with Stage III/IV disease observed for most loci emphasize the importance for future studies to include detailed sub-phenotype information. Functional studies in relevant tissues are needed to understand the effect of the variants on downstream biological pathways.

## Introduction

Endometriosis is a common, estrogen-dependent, inflammatory condition associated with chronic pelvic pain, subfertility and dysmenorrhoea (Giudice and Kao, 2004; Berkley *et al.*, 2005). Its estimated prevalence rates range from 5–10% in women of reproductive age in the general population to 35–50% among women with chronic pelvic pain and subfertility (Eskenazi and Warner, 1997). The causes of the condition are largely unknown, but are likely to be complex, involving multiple environmental and genetic factors. Based on a study of 3096 twins, the heritability of endometriosis, the proportion of disease variance due to genetic factors, has been estimated at around 52% (Treloar *et al.*, 1999).

To elucidate causal genetic variants underlying endometriosis, many investigators have used so-called ‘candidate gene’ study approaches over the past decades. Candidate gene study approaches are based on a hypothesis, which can be biological or positional. In biological candidate gene studies, genes with an inferred biological relevance to the disease are selected and genetic variants in these genes are tested for association with the disease of interest. In positional candidate gene studies, variants and genes are selected on the basis of prior evidence that a specific genomic region is implicated, for example through hypothesis-free linkage studies described below. Few such positional candidate gene studies have been performed in endometriosis (Treloar *et al.*, 2007; Zhao *et al.*, 2008; Lin *et al.*, 2011). Biological candidate gene studies in endometriosis have been abundant and, similar to other complex diseases, it is fair to say that they have been generally unsuccessful, with limited replicated results (Montgomery *et al.*, 2008; Rahmioglu *et al.*, 2012). Reasons for the general failure of candidate gene studies to elucidate genetic mechanisms in complex disease are clear: (i) they are based on a biological hypothesis that may not be true; (ii) only one or a few genes in a relevant biological pathway are typically tested; (iii) usually only a few variants in a gene are tested, and no attempt is made to comprehensively cover the gene; (iv) cases and controls used are often poorly defined, or definitions vary and (v) sample sizes are usually inadequate to detect the effect sizes that are expected for variants influencing a complex trait (Zondervan *et al.*, 2002).

Rather than following hypotheses with little *a priori* probability of success, many investigators have turned to hypothesis-free approaches to uncover genetic variants underlying disease on a genome-wide scale. There are two such hypothesis-free approaches: (i) family-based linkage studies and (ii) population-based genome-wide association studies (GWASs). Family-based linkage studies are aimed at identifying genomic regions harbouring genetic variants that are typically rare in the general population, and responsible for the aggregation of a disease in families with multiple affected individuals.

Linkage studies have been very successful in identifying genetic variants responsible for rare, monogenic disorders, in which a mutation confers a very high risk of disease, but they have not generally been very successful in complex diseases, where genetic variants confer modest increases in susceptibility to disease. Exceptions are scenarios in which families are identified with strong familial aggregation of what is generally considered a complex disease. A well-known example of such a scenario is the identification of the *BRCA* genes habouring rare variants conferring high risk of familial breast and ovarian cancer (Easton, 1999; Pal *et al.*, 2005; Campeau *et al.*, 2008). In 2005, the International Endogene Study published evidence of significant linkage of endometriosis to chromosome 10q26 from analysis of 1176 affected sister-pair families (Treloar *et al.*, 2005); subsequent fine-mapping association analyses of 10q26 suggested possible association of common variants near *CYP2C19* (Painter *et al.*, 2011a, b), but rare variants that could explain the linkage signal remain to be identified. In 2007, the same study identified significant linkage to chromosome 7p13-15 in a sub-analysis of 248 families with more than 3 affected members (Zondervan *et al.*, 2007), suggesting the presence of one or more rare variants conferring high risk; analyses are ongoing to identify the variants responsible (Lin *et al.*, 2011).

**Figure 1:**
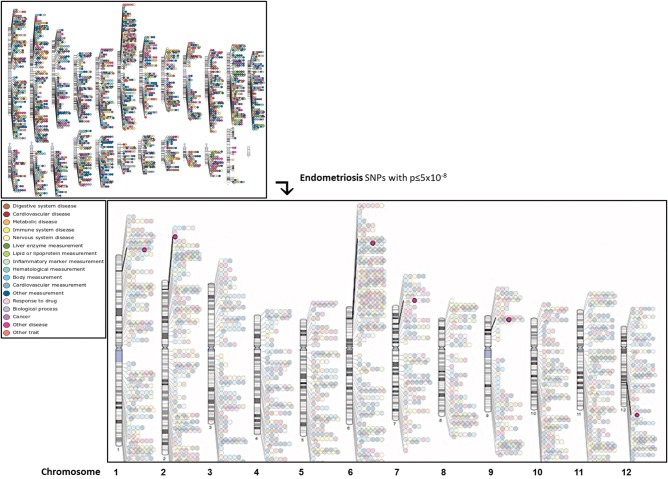
Schematic overview of all genome-wide associations (*P* ≤ 5 × 10^−8^) across all chromosomes (small window) and endometriosis associations (large window), presented in 17 trait categories (colour coded), generated from the NHGRI GWA Catalog (Hindorff *et al.*, 2009). Available at: www.genome.gov/gwastudies (accessed 11 October 2013).

Population-based GWASs are founded on the principle that common diseases, such as endometriosis, are caused by genetic variants that are common themselves (common disease-common variant hypothesis). Thus, they can be seen as a complementary approach to linkage studies, which are designed to find rare variants contributing to familial disease. The principle of GWAS is simple, in that a set of several 100 000 common single nucleotide polymorphisms (SNPs or single DNA base pair changes), selected to provide the maximum coverage of the genome, is genotyped in a large set of cases and controls and their frequencies are compared between the two groups. This SNP selection is based on ‘linkage disequilibrium’ (LD) between SNPs, which is the non-random correlation of genetic variants in a population that exists due to shared ancestry of chromosomes. SNPs selected to ‘tag’ (represent) parts of the genome can be used to predict the allelic status of a genetic variant nearby because of shared ancestry of that particular genomic segment. Thus, several 100 000 s of tagSNPs can be used to provide information on most of the ∼10 million common SNPs present in the human genome. In the past 5 years, GWAS have proved to be very successful in identifying many common genetic variants associated with complex disease, as demonstrated by the NHGRI Catalog of published GWAS (http://www.genome.gov/gwastudies/; Fig. 1) (Hindorff *et al.*, 2009; Visscher *et al.*, 2012). The current catalogue includes association results for 11 751 SNPs from 1738 publications (November 2013). Notably, 88% of identified SNPs are either in inter-genic regions (43%) or located in intronic (non-coding) regions (45%) of genes, demonstrating that the interpretation of the signals typically requires further studies exploring the functionality of these regions (Hindorff *et al.*, 2009). Indeed, the ENCODE project has shown that ∼80% of non-coding regions are likely to have functionality regulating gene expression (ENCODE Project Consortium, 2012).

### GWAS of endometriosis

In 2010, the first endometriosis GWAS was published on a Japanese dataset of 1907 cases and 5292 controls (Uno *et al.*, 2010), providing genome-wide significant evidence for association of a variant in *CDKN2B-AS1* (cyclin-dependent kinase inhibitor 2B antisense RNA) [rs10965235; odds ratio (OR) = 1.44 (95% confidence interval (CI): 1.30–1.59), *P* = 5.57 × 10^−12^]. This publication was quickly followed by that of a smaller Japanese GWAS of 696 cases and 825 controls that did not find a significant signal (Adachi *et al.*, 2010). The first GWAS in women of European ancestry was published in 2011 by the International Endogene Consortium (IEC), involving 3194 surgically confirmed cases and 7060 controls from Australian and UK datasets, with independent replication in a US dataset of 2392 cases and 2271 controls (Painter *et al.*, 2011a, b). This study provided genome-wide significant evidence for an inter-genic locus on chromosome 7 [rs12700667; OR = 1.22 (95% CI: 1.13–1.32), *P* = 1.4 × 10^−9^], and combined with the published results from Uno *et al.* (2010), for association with an SNP near *WNT4* (wingless-type MMTV integration site family member 4) [rs7521902; OR = 1.19 (95% CI: 1.12–1.27), *P* = 4.2 × 10^−8^]. Both signals showed much stronger evidence for association with moderate-to-severe [revised American Fertility Society (rAFS) Stage III/IV] endometriosis: rs12700667, OR = 1.38 (95% CI: 1.24–1.53); rs7521902: OR = 1.25 (95% CI: 1.12–1.27). The IEC study could not replicate the signal for rs10965235 seen in the Japanese GWAS by Uno *et al.* (2010), as this variant was monomorphic (non-variable) in individuals of European ancestry, nor could they find association with variants nearby. In 2012, a meta-analysis of summary results, combining the evidence from the Japanese and IEC GWAS datasets, was conducted. This analysis confirmed the three loci published by the two original papers, and provided evidence for a further four (Nyholt *et al.*, 2012): rs10859871 near *VEZT* [OR = 1.20 (95% CI: 1.14–1.26); *P* = 5.1 × 10^−13^]; rs4141819 in an inter-genic region on 2p14 [OR = 1.15 (95% CI: 1.09–1.21), *P* = 8.5 × 10^−8^]; rs7739264 near *ID4* (inhibitor of DNA binding 4) [OR = 1.17 (95% CI: 1.11–1.23), *P* = 3.6 × 10^−10^] and rs1537377 near *CDKN2B-AS1* [OR = 1.15 (95% CI: 1.10–1.21), *P* = 2.4 × 10^−9^].

Since the Nyholt *et al.* (2012) meta-analysis, a fourth GWAS in women of European ancestry was published, involving 2019 cases and 14 471 controls from the USA (Albertsen *et al.*, 2013). This large study reported lack of significant replication of the IEC signal for rs12700667 on chromosome 7 (*P* = 0.12), and found two genome-wide significant signals: (i) near *WNT4* (rs2235529; *P* = 8.65 × 10^−9^), tagging the same locus for which association was reported by the IEC/Japanese meta-analyses (*r*^2^ = 0.66 with rs7521902) and (ii) a novel intergenic signal 280Kb upstream of *RND3-RBM43* (rs1519761; *P* = 4.70 × 10^−8^). In addition, two replication studies were published, which genotyped some of the implicated SNPs in women of European ancestry. A study involving 1129 surgically confirmed cases and 831 controls from Belgium (Sundqvist *et al.*, 2013) did not find significant evidence either for rs12700667 on chromosome 7 (*P* = 0.46), or for rs7521902 in *WNT4* (*P* = 0.17). A second replication study in 305 surgically confirmed cases and 2710 controls from Italy (Pagliardini *et al.*, 2013) also could not replicate rs12700667 (*P* = 0.80) but did find significant evidence for *WNT4* (rs7521902, *P* = 5.6 × 10^−3^), *FN1* (fibronectin 1) (rs1250248, *P* = 9.0 × 10^−3^) and an SNP in *CDKN2B-AS1* (rs1333049, *P* = 1.7 × 10^−3^). Results in these more recent papers have been interpreted as showing evidence for heterogeneity in the genetic loci underlying endometriosis in different populations, even when sampled from women with similar ethnic ancestry.

### Sources of heterogeneity

#### Genetic population heterogeneity

Association studies assume that the allele frequency differences of genetic variants (SNPs) observed between cases and controls reflect genetic factors underlying the disease. Common SNPs (population allele frequency >0.05) genotyped throughout the genome for the association studies are selected on the basis of LD. Different patterns of LD exist between populations due to differing mutational events and selection pressures experienced by different populations throughout history. The International HapMap Project has determined LD patterns of SNPs across the genome through characterizing the genetic variants, and their frequencies and correlations, in DNA samples from 11 different ethnic populations (The International HapMap Consortium, 2005, 2010). This has enabled the discovery of common genetic risk variants indirectly through testing tagSNPs that are highly predictive of the status of other SNPs, providing very dense coverage of the genome without genotyping all the variants in the region of interest, in each reference population.

Due to population history differences, some of the disease risk variants implicated in one ethnic population may not be associated, or associated with a greater or lesser extent, with disease risk in another population. This problem is worse for more recent mutations, deleterious mutations that are found at lower frequencies and which tend not to be shared between populations (making most of the rare mutations private to different ethnic populations), or those that affect traits with reduced reproductive fitness/fertility. Arguably, endometriosis could be an example of such a trait, although it is unknown whether endometriosis would have affected the fertility of women in early reproductive years in previous centuries.

#### Phenotypic heterogeneity: disease definition

Variability in phenotypic characterization of endometriosis cases between studies is likely to contribute to the heterogeneity in findings, and may result in genetic risk variants going undetected due to dilution of the strength of association. Variability may arise because of differences in the proportions of endometriosis sub-phenotypes such as endometrioma, recto-vaginal and peritoneal disease, or different frequencies of rAFS stage (American Society for Reproductive Medicine, 1997), which may have different genetic origins. In addition, some GWASs have included endometriosis cases that were diagnosed through methods other than the gold standard of laparoscopic surgery, such as ultra-sound imaging or clinical symptoms. Furthermore, women with endometriosis in the studies may vary according to pain or subfertility phenotypes which, if available at all, are likely to have been assessed through different, non-standardized, means. Also, the route to diagnosis can vary greatly between clinics and countries, due to referral patterns and cultural and socio-economic differences, all of which could introduce heterogeneity in terms of the type of cases that are included in the studies.

Given the suggested heterogeneity in genetic signals from endometriosis GWAS and replication studies, and the potential for genetic and phenotypic variability to influence results, we investigated the heterogeneity and consistency of results across all published GWAS and replication datasets from Australia, Belgium, Italy, Japan, the UK and the USA through meta-analyses of 11 SNPs in 9 loci that reached genome-wide significance in at least 1 study.

## Methods

### Descriptions of GWAS and replication studies and populations

For this meta-analysis, we identified all the GWAS of endometriosis and replication studies published up to 1 December 2013. A systematic literature search in PubMed for English language publications was performed using the terms ‘endometriosis’ and ‘GWAS’ and/or ‘replication’. Table I shows all four GWAS and four replication datasets included in the meta-analysis, and their population origins. One GWAS dataset was of Japanese origin, and consisted of 1423 case and 1318 controls obtained from the BioBank Japan (Uno *et al.*, 2010). Individuals were genotyped on the Illumina 550 K BeadChip array. Cases were diagnosed through the presence of multiple clinical symptoms, physical examinations and/or laparoscopic surgery; no information on disease stage or other sub-phenotypic disease data was available. The remaining three GWAS datasets are of European ancestry, with all cases laparoscopically confirmed: 2270 (40% rAFS Stage III/IV) cases and 1870 controls from Australia (‘QIMR’); 924 (49% Stage III/IV) cases and 5190 controls from the UK (‘OX’) (Painter *et al.*, 2011a), and 2019 (42% Stage III/IV) cases and 14 471 controls from the USA (‘UTAH’) (Albertsen *et al.*, 2013). The control sets in all GWAS datasets were population based and unscreened for endometriosis cases. The IEC Australian and UK datasets were genotyped on Illumina 610/670 K and 670 K/1M chips, respectively, while the US dataset was genotyped using Illumina OmniExpress 730 K BeadChip. The IEC datasets were imputed up to 1000 Genomes Pilot reference panel (B36, June 2010). From the SNPs included in this meta-analysis, only rs1333049 was an imputed SNP in the QIMR Australian dataset.

**Table I:** Summary of the eight published endometriosis GWAS and replication studies included in the meta-analysis.

Cohort	Ancestry	No. of cases	No. of stage III/IV cases^a^	No. of controls	References
OX GWAS	European (UK/USA/EU)	924	454	5190	Painter *et al.* (2011a)
QIMR GWAS	European (Australia)	2270	908	1870	Painter *et al.* (2011a)
Utah GWAS	European (USA)	2019	848	14 471	Albertsen *et al.* (2013)
NHS II replication	European (USA)	2392	No stage info	2271	Painter *et al.* (2011a)
Pagliardini replication	European (Italy)	305	220	2710	Pagliardini *et al.* (2013)
Sundqvist replication	European (Belgium)	1129	429	831	Sundqvist *et al.* (2013)
*Total European ancestry*		*9039*	*2859*	*27* *343*	
BBJ GWAS	Japanese	1423	No stage info	1318	Uno *et al.* (2010)
BBJ replication	Japanese	1044	No stage info	4017	Uno *et al.* (2010)
*Total Japanese ancestry*		*2467*		*5335*	
Total		11 506	2859	32 678	

^a^rAFS III and IV disease only. OX, Oxford University; QIMR, Queensland Institute of Medical Research; NHS II, Nurses' Health Study II; BBJ, BioBank Japan.

Of the four replication studies, one was conducted in women of Japanese, and three in women of European ancestry (Table I). The datasets comprised: (i) 2392 self-reported surgically diagnosed cases (no disease stage information) and 2271 unscreened population controls from the Nurse's Health Study II (NHS II) in the USA (Painter *et al.*, 2011a); (ii) 305 laparoscopically confirmed cases (72% Stage III/IV cases) and 2710 population-based controls (90% unscreened for endometriosis) from Italy (Pagliardini *et al.*, 2013); (iii) 1129 laparoscopically confirmed cases (38% stage III/IV cases) and 831 laparoscopy-negative controls from a subfertility clinic population in Belgium (Sundqvist *et al.*, 2013) and (iv) 1044 cases (no disease stage information) and 4017 unscreened population controls from BioBank Japan (Uno *et al.*, 2010); 653 of these 1044 cases had been used in a small GWAS of 696 cases and 825 controls, which had not provided a genome-wide significant result (Adachi *et al.*, 2010).

### Meta-analysis of genome-wide significant results across the studies

We performed a meta-analysis of association results for SNPs passing the *P*-value threshold < 5 × 10^−8^ (‘genome-wide significance’) in at least one of the studies, across all of the endometriosis GWAS and replication datasets, including a total of 11 506 cases and 32 678 controls, employing a fixed-effect model in the first instance, using GWAMA software (Mägi and Morris, 2010). ORs and 95% CIs and the sample size of the studies for each SNP were used as input to the model. SNPs that maintained a *P*-value < 5 × 10^−8^ on meta-analysis were considered genome-wide significant. Given the evidence for a substantially increased contribution of genetic factors to moderate/severe (rAFS Stage III/IV) compared with minimal/mild (rAFS Stage I/II) endometriosis (Painter *et al.*, 2011a, b), additional meta-analyses were conducted on ‘Stage III/IV enriched’ and ‘Stage III/IV only’ datasets. The ‘Stage III/IV-enriched’ analysis included association results from QIMR, OX, UTAH, Pagliardini and Sundqvist Stage III/IV cases (*n* = 2859) versus controls, combined with all-stage endometriosis versus controls association results from the BBJ, BBJ replication and NHS II replication datasets (for which stage information on cases was not available). Thus, the ‘Stage III/IV-enriched’ analysis was based on 7718 cases and 32 678 controls. The Stage III/IV-only analysis included association results from 2859 Stage III/IV cases (QIMR, OX, UTAH, Pagliardini and Sundqvist) versus 32 678 controls.

Heterogeneity of allelic effects across studies was examined using the Cochran's *Q* test (Cochran, 1954). Between-study heterogeneity was indicated by *Q* statistic *P* values < 0.1 (Ioannidis *et al.*, 2007), as well as the *I*^2^ index, which indicates the percentage of variance attributable to heterogeneity (Higgins and Thompson, 2002). Given that the nine loci represented nine independent tests, a Bonferroni correction (0.05/9) was applied to the threshold for significant evidence of heterogeneity (*P* < 0.005). Meta-analysis of SNPs that showed evidence of effect heterogeneity was also carried out using the Han and Eskin random-effects model, which increases power to detect associations under heterogeneity, implemented in METASOFT software (Han and Eskin, 2011). Beta effect sizes, standard errors and sample sizes for each SNP were used for input to the model.

The variance explained by the established loci associated with endometriosis was calculated by transformation of dichotomous disease risk onto a continuous liability scale that assumes a disease prevalence rate and a multiplicative model (Wray *et al.*, 2010; Morris *et al.*, 2012).

### Investigation of other reported GWAS associations in endometriosis loci

For each endometriosis SNP included in the meta-analysis, we determined the chromosomal band it is located in, using UCSC build 19. We then extracted all GWAS that reported genome-wide significant SNP associations with any disease or trait for the respective chromosomal segments from the NHGRI resource (http://www.genome.gov/gwastudies/) (Hindorff *et al.*, 2009). We determined the distance between each endometriosis SNP and SNPs associated with other diseases/traits in each chromosomal segment, and assessed LD (correlation, *r*^2^) between each SNP pair in the Caucasian and Japanese populations using the 1000 Genomes Pilot CEU and JPT data reference panels, implemented in Haploview (Barrett *et al.*, 2005). An *r*^2^ > 0.2 was used as the threshold for SNPs that were in moderate LD (1000 Genomes Project Consortium, 2010).

## Results

### Consistency and heterogeneity of genetic associations between studies and populations

**Figure 2:**
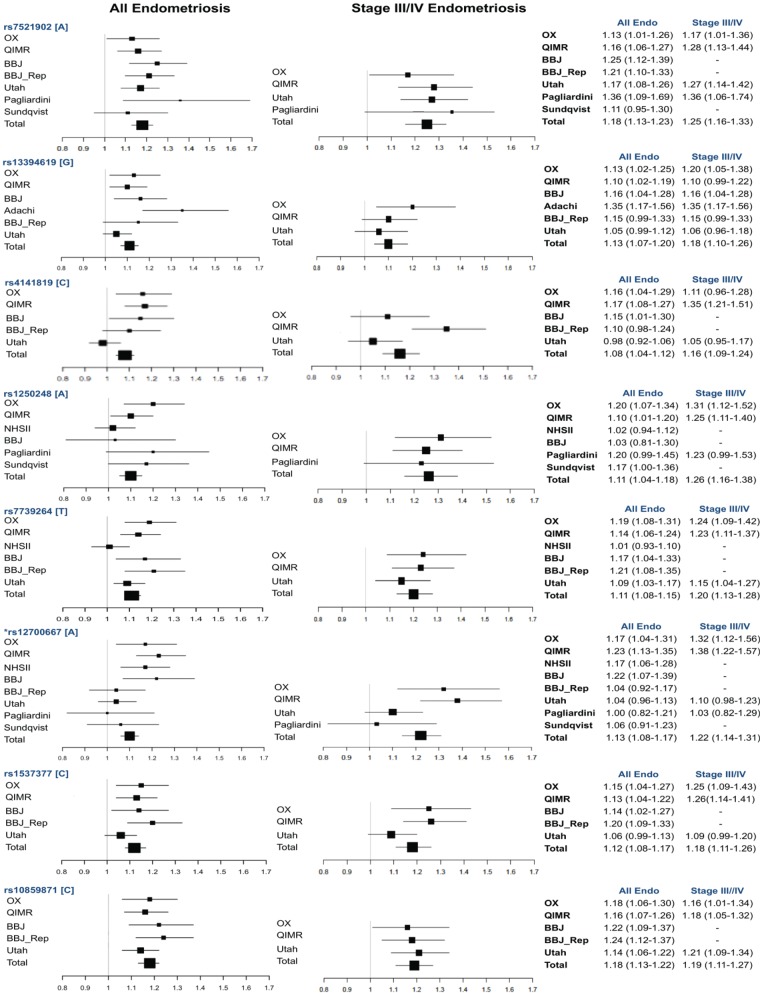
Forest plots showing the effects of risk alleles for SNPs in six loci reaching genome-wide significance for association with all endometriosis and two loci reaching borderline genome-wide significance with only Stage III/IV cases in the meta-analysis. BBJ_Rep, BioBank Japan replication.

Of the 11 SNPs in 9 loci associated with endometriosis in at least one of the 8 studies, the meta-analysis showed genome-wide significant evidence (*P* < 5 × 10^−8^) for seven SNPs (six loci), with consistent directions of effect across studies and populations (Table II, Fig. 2). These included rs7521902 near *WNT4* [OR = 1.18 (95% CI: 1.13–1.23) *P* = 1.8 × 10^−15^]; rs13394619 in *GREB1* (growth regulation by estrogen in breast cancer 1) [OR = 1.13 (95% CI: 1.07–1.20), *P* = 2.9 × 10^−8^]; rs7739264 near *ID4* [OR = 1.11 (95% CI: 1.08–1.15), *P* = 6.2 × 10^−10^]; both rs12700667 [OR = 1.10 (95% CI: 1.06–1.14), *P* = 1.6 × 10^−9^] and rs7798431 [OR = 1.13 (95% CI: 1.09–1.18), *P* = 5.4 × 10^−9^] in an inter-genic region on chromosome 7 (correlation *r*^2^ between SNPs = 0.8); rs1537377 near *CDKN2B-AS1* [(OR = 1.12 (95% CI: 1.08–1.17), *P* = 1.0 × 10^−8^] and rs10859871 [OR = 1.18 (95% CI: 1.13–1.22), *P* = 4.8 × 10^−15^] in *VEZT*. Interestingly, rs1250248 in *FN1* showed a strong association with Stage III/IV disease (*P* = 8.0 × 10^−8^), had consistent directions of effect across all studies and populations, but just fell short of the genome-wide significance threshold.

**Table II:** Results of the meta-analysis of the 11 published SNPs genome-wide significantly associated with endometriosis in at least one study.

Chr	SNP	Position (HG19)	RA	RAF in CEU	RAF in JAP	Case selection^e^	No. of studies	Meta-analysis results					Nearest gene
								*P*_meta_^b^	Direction	*Q* P_het_	*I*^2^**	OR_Meta_ (95% CI)	(distance)
1	rs7521902^a^	22490474	A	0.23	0.33	All	7	1.8 × 10^−15^	+++++++	0.83	0	1.18 (1.13–1.23)	*WNT4*
						III/IV enriched	7	2.7 × 10^−17^	+++++++	0.81	0	1.23 (1.17–1.28)	(21 kb)
						III/IV only	4	1.8 × 10^−10^	++++	0.80	0	1.25 (1.16–1.33)	
2	rs13394619^c^	11727257	G	0.53	0.42	All	6	4.5 × 10^−8^ (2.9 × 10^−8^)	++++++	0.04*	56.1	1.13 (1.07–1.20)	*GREB1*
						III/IV enriched	6	3.5 × 10^−8^	++++++	0.13	39.8	1.15 (1.09–1.20)	(0)
						III/IV only	3	2.1 × 10^−3^	+++	0.34	25.6	1.18 (1.11–1.24)	
2	rs4141819	67864425	C	0.27	0.22	All	5	2.1 × 10^−4^ (8.8 × 10^−6^)	++++−	0.004*	70.6	1.08 (1.04–1.12)	Intergenic
						III/IV enriched	5	2.5 × 10^−5^ (9.2 × 10^−8^)	++++−	0.02*	66.0	1.15 (1.09–1.21)	(*ETAA1:*
						III/IV only	3	6.9 × 10^−6^ (1.0 × 10^−6^)	+++	0.003*	81.9	1.16 (1.09–1.24)	227 kb)
2	rs1250248	216286843	A	0.21	0.03	All	6	1.1 × 10^−4^	++++++	0.21	23.0	1.11 (1.04–1.18)	*FN1*
						III/IV enriched	6	1.3 × 10^−5^	++++++	0.86	0	1.13 (1.07–1.19)	(0)
						III/IV only	3	8.0 × 10^−8^	+++	0.86	0	1.26 (1.16–1.38)	
2	rs6734792	151624632	C	0.38	0.32	All	4	9.7 × 10^−5^ (2.2 × 10^−6^)	+++?	0.003*	78.3	1.10 (1.06–1.16)	Intergenic
						III/IV enriched	4	5.5 × 10^−3^	++++	0.13	49.8	1.08 (1.02–1.15)	(*RND3*:
						III/IV only	3	6.5 × 10^−5^ (2.7 × 10^−5^)	+++	0.002*	84.4	1.10 (1.05–1.15)	281 kb)
6	rs7739264	19785338	T	0.55	0.77	All	6	1.9 × 10^−10^ (6.2 × 10^−10^)	++++++	0.05*	50.9	1.11 (1.08–1.15)	*ID4*
						III/IV enriched	6	6.7 × 10^−10^ (3.1 × 10^−10^)	++++++	0.02*	61.0	1.14 (1.10–1.19)	(52 kb)
						III/IV only	3	1.2 × 10^−8^	+++	0.56	0	1.20 (1.13–1.28)	
7	rs12700667	25901389	A	0.76	0.20	All	8	1.9 × 10^−9^ (1.6 × 10^−9^)	++++++++	0.05*	51.0	1.13 (1.08–1.17)	Intergenic
						III/IV enriched	8	7.0 × 10^−11^ (4.2 × 10^−11^)	++++++++	0.02*	57.9	1.17 (1.11–1.22)	(*NFE2L3:*
						III/IV only	4	4.5 × 10^−8^ (3.6 × 10^−8^)	++++	0.02*	69.5	1.22 (1.14–1.31)	290 Kb)
7	rs7798431^d^	25860562	G	0.79	0.49	All	5	5.4 × 10^−9^	+++++	0.14	37.0	1.13 (1.09–1.18)	Intergenic
						III/IV enriched	5	8.5 × 10^−10^	+++++	0.15	40.8	1.19 (1.13–1.26)	(*NFE2L3:*
						III/IV only	3	9.7 × 10^−8^	+++	0.10	58.3	1.24 (1.14–1.33)	331 Kb)
9	rs1537377	22169450	C	0.40	0.39	All	5	1.0 × 10^−8^	+++++	0.30	18.8	1.12 (1.08–1.17)	*CDKN2B-*
						III/IV enriched	5	5.8 × 10^−12^	+++++	0.25	23.8	1.18 (1.13–1.23)	*AS1*
						III/IV only	3	8.1 × 10^−8^ (2.3 × 10^−7^)	+++	0.09*	58.1	1.18 (1.11–1.26)	(48 Kb)
9	rs1333049	22125253	G	0.54	0.02	All	3	0.25 (0.12)	++−	0.01*	77.3	1.04 (0.98–1.10)	*CDKN2B-*
						III/IV enriched	3	0.05 (0.03)	++−	0.03*	70.9	1.08 (1.00–1.17)	*AS1*
						III/IV only	2	0.55 (0.60)	++	0.47	0	1.03 (0.94–1.12)	(4 Kb)
12	rs10859871	95711626	C	0.30	0.29	All	5	4.8 × 10^−15^	+++++	0.69	0	1.18 (1.13–1.22)	*VEZT*
						III/IV enriched	5	3.0 × 10^−13^	+++++	0.94	0	1.20 (1.15–1.27)	(17Kb)
						III/IV only	3	6.8 × 10^−7^	+++	0.88	0	1.19 (1.11–1.27)	

RA, risk allele; RAF, risk allele frequency; CEU, Caucasian; JPT, Japanese; OR, odds ratio; 95% CI, 95% confidence interval.

^a^Also, in moderate LD with rs2235529 (*r*^2^ = 0.7), which was identified as genome-wide significant in the Utah dataset (Albertsen *et al.*, 2013).

^b^Meta-analysis *P*-value is reported from the fixed-effects model as implemented in GWAMA software (Mägi and Morris, 2010). Where significant heterogeneity is detected, the results from the random-effects model optimized to detect associations under heterogeneity are reported in parenthesis as implemented in MetaSoft software (Han and Eskin, 2011).

^c^The meta-analysis for rs13394619 includes results published in Adachi *et al.* (2010), obtained from combined analysis of 500 K and 6.0 arrays in 696 cases and 825 controls. Moreover, rs13394619 was an imputed SNP in the QIMR Australian dataset.

^d^Rs12700667 and rs7798431 are highly correlated SNPs with *r*^2^ = 0.8; rs7798431 does not represent an independent signal (Painter *et al.*, 2011a).

^e^For each SNP, the results from three separate meta-analyses are reported including: (i) all endometriosis cases to controls, (ii) Stage III/IV enriched cases to controls and (iii) Stage III/IV only cases to controls (see Methods).

*Significant heterogeneity detected by Cochran's *Q* test, *P* value < 0.1.

**In addition to Cochran's *Q* test, *I*^2^ heterogeneity index is given which indicates the percentage of variance attributable to heterogeneity.

The results show that genetic heterogeneity between populations is not governing these six confirmed endometriosis loci. For three of the seven SNPs, the between-study heterogeneity *Q* test *P*-value was borderline significant (0.01 < *P* < 0.1) when considering that nine independent tests were conducted; however, the results from the random-effects model allowing for heterogeneity showed negligible difference from those obtained using fixed-effect meta-analysis, and they reached genome-wide significance under both models, demonstrating the lack of evidence for significant heterogeneity of signals between studies (Table II). One of the directionally consistent loci is rs12700667, in an inter-genic region of high LD (48Kb *r*^2^ > 0.8) with rs7798431 on chromosome 7. The previously reported lack of significant association of rs12700667 and rs7798431 variants in the individual Belgian (Sundqvist *et al.*, 2013), Italian (Pagliardini *et al.*, 2013) and Utah datasets (Albertsen *et al.*, 2013) is therefore likely to be due to stochastic fluctuations that typically can be seen between individual datasets. It also highlights an important point about the need to interpret study results as probabilities, and demonstrates the complex issue of what (lack of) ‘replication’ means when a fixed *P*-value threshold such as *P* < 0.05 is used. A variant with an association *P* > 0.05 does not imply the variant is not associated; rather, it means that, assuming the variant has no effect, if we conducted the same study 100 times, we would see the result in at least 5% of instances.

Two inter-genic loci, both on chromosome 2, showed evidence of heterogeneity (*P* < 0.005) between studies: rs4141819 (closest gene: *ETAA1*) and rs6734792 (closest gene: *RND3*). Random-effects analyses substantially increased the significance of rs4141819, most notably for association with Stage III/IV disease (enriched analysis: *P* = 9.2 × 10^−8^), but had little effect on association with rs6734792 (all endometriosis: *P* = 2.2 × 10^−6^).

**Figure 3:**
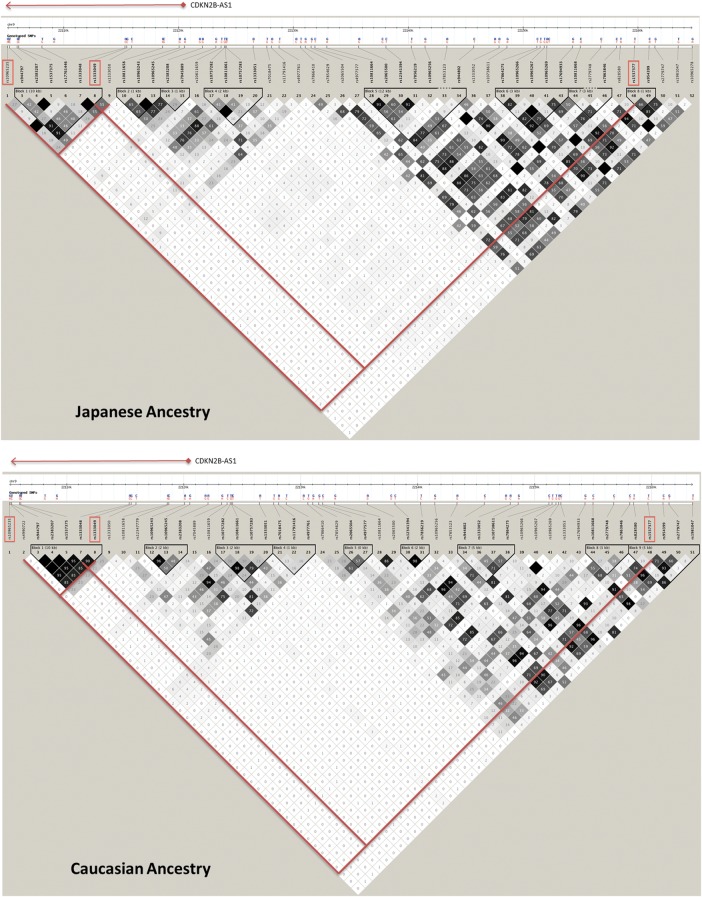
Linkage disequilibrium structure for the region containing rs1537377, rs1333049 and rs10965235 in/near CDKN2BAS on chromosome 9 in individuals of European ancestry (bottom panel) and of Japanese ancestry (top panel) (*Source:* http://hapmap.ncbi.nlm.nih.gov).

An intriguing genomic region, because of the differences in allele frequencies and LD structure between individuals of Japanese and European ancestry, is the *CDKN2B-AS1* locus on chromosome 9 (Table II, Fig. 3). In the first Japanese GWAS of endometriosis by Uno *et al.* (2010), the authors reported association with rs10965235 in *CDKN2B-AS1* gene. This SNP, however, is monomorphic in individuals of European ancestry, and therefore direct replication of this signal was not possible in datasets of this ancestry. However, in the Nyholt *et al.* meta-analysis of the BBJ, QIMR and OX datasets, genome-wide significant association was reported across these datasets for rs1537377 [*P* = 1.0 × 10^−8^, OR = 1.22 (95% CI: 1.14–1.30], 55 kb away from rs10965235. LD (correlation) between rs10965235 and rs1537377 in the Japanese HapMap reference population is very low (*r*^2^ = 0.01) (Fig. 3). Nyholt *et al.* (2012) conducted conditional association analysis in the Japanese dataset, providing suggestive evidence for rs1537377 to be a second, independent, risk variant in the *CDKN2B-AS1* region for endometriosis. Our present meta-analysis certainly suggests that rs1537377 is a risk variant across Japanese and European ancestry datasets, showing the strongest association with Stage III/IV disease (Stage III/IV enriched: *P* = 5.8 × 10^−12^). In the Italian replication study, Pagliardini *et al.* genotyped rs1333049 in the *CDKN2B-AS1* region, based on its location in the same Japanese LD block as rs10965235 (Fig. 3) and on its common frequency in the Italian population [HapMap minor allele frequency in Tuscans in Italy (TSI) = 0.48]. In our meta-analysis including unpublished data from UK (OX GWAS) and Australian (QIMR GWAS) datasets, rs1333049 did not reach genome-wide significance (Stage III/IV enriched: *P* = 0.05).

Assuming a population prevalence for endometriosis of 8% (Zondervan *et al.*, 2001, 2002; Missmer *et al.*, 2004), these nine loci together account for 1.67% of variance in ‘all’ endometriosis susceptibility (Wray *et al.*, 2010). Therefore, they currently have no immediate role in risk prediction.

### Results for clinical sub-phenotypes

Of the seven genome-wide significantly associated SNPs in our meta-analysis (six loci), six SNPs (five loci) showed an increasing effect size (OR) as the proportion of cases with Stage III/IV disease included in analyses increased from all endometriosis to ‘Stage III/IV-enriched’ to ‘Stage III/IV-only’ (Table II). Figure 2 shows forest plots for the results of these loci from individual studies, for association with all endometriosis and Stage III/IV disease, highlighting the point that for most loci, effect sizes were greater for association with Stage III/IV disease. These results imply that the loci are likely to be implicated predominantly in the development of moderate/severe disease. The most striking observation of an association that became close to genome-wide significance when limiting cases to the much smaller subset with known Stage III/IV disease (*n* = 2859) is rs1250248 in *FN1*, which had an association OR = 1.11 (95% CI: 1.04–1.18, *P* = 1.1 × 10^−4^) with all endometriosis, and an OR of 1.27 (95% CI: 1.16–1.38), *P* = 8.0 × 10^−8^) with Stage III/IV disease.

### Current understanding of biological mechanisms of the genetic loci

**Figure 4:**
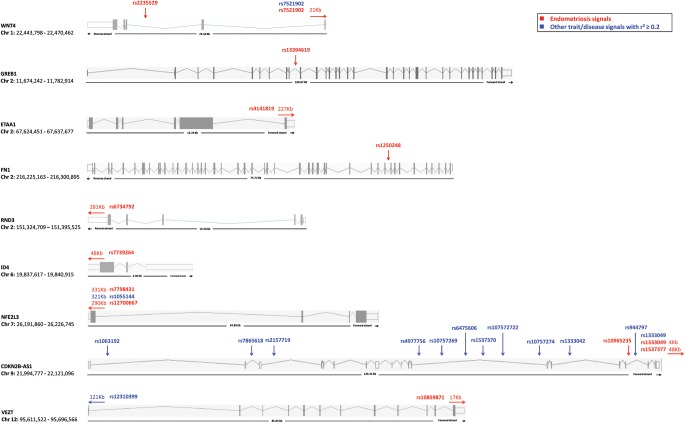
Diagrams showing the 9 genes closest to each of the 11 endometriosis SNPs included in the meta-analysis. Exons (coding regions) are presented with grey coloured boxes; lines between the exons present introns (non-coding genic regions); empty white boxes at the ends of the genes represent the 3′ and 5′ UTR regions. Each endometriosis SNP is illustrated in red, along with its distance to the gene where relevant (red arrows). SNPs genome wide associated with other trait/disease associations, that are in linkage disequilibrium (*r*^2^ > 0.2) with any of the endometriosis SNPs are illustrated in blue. See Supplementary data, Table SI for a complete list of all published SNP associations for these genomic regions including independent signals.

Figure 4 depicts the genes nearest to the nine loci (eleven SNPs) analysed in our meta-analysis, showing the location of each SNP in relation to the gene and its functional structures. Also shown is the location of known SNPs within the gene reported to be genome wide associated with other traits and diseases (source: NIHGR GWAS database, http://www.genome.gov/gwastudies/; November 2013), that are in LD (*r*^2^ > 0.2) with any of the endometriosis SNPs. A comprehensive list of all published GWAS variants that are located in the genes but do not correlate (*r*^2^ < 0.2) with endometriosis SNPs, or are in LD with endometriosis variants outside genes, is provided in Supplementary data, Table SI.

A total of four SNPs in three genetic loci, rs12700667/rs7798431 on chromosome 7p15.2, rs4141819 on 2p14 and rs6734792 on 2q23.3 are in inter-genic regions with no known genes within 200 kb. Recent evidence from the ENCODE project has strongly suggested that these regions are likely to play an important role in gene transcription regulation (The ENCODE Consortium, 2012), and variants located in these regions may be involved in *cis*- or *trans*-regulation of genes distantly located. Although we report the genes nearest to the associated variants (Fig. 4), this does not necessarily imply that it is the regulation of these genes that is affected by the variants. Further studies on the effect of the variants on gene expression (eQTL studies) are required to understand how they affect biological pathways.

Intergenic rs12700667 on 7p15.2 is in high LD with rs1055144 (*P* = 1.0 × 10^−24^; *r*^2^ = 0.5, 1000G pilot CEU data; Fig. 4) that was previously associated with waist-to-hip ratio adjusted for body mass index (WHRadjBMI), a measure of fat distribution, in an independent GWAS of ∼190 000 individuals (Heid *et al.*, 2010). The 7p15.2 region contains several potential candidate genes. *NFE2L3* [Nuclear factor (erythroid-derived 2-like 3] is a transcription factor suggested to be involved in cell differentiation, inflammation and carcinogenesis (Chevillard and Blank, 2011). *NFE2L3* mRNA levels were found to be up-regulated in human breast cancer cells (Rhee *et al.*, 2008) and testicular carcinoma tissue samples (Almstrup *et al.*, 2007). Also, interferon-gamma has been shown to increase *NFE2L3* mRNA levels in human uterine endothelial cells (Kitaya *et al.*, 2007). However, its potential role in endometriosis aetiology remains to be discovered. A second locus of interest in this region is a microRNA, *miRNA_148a*, with a purported role in the Wnt/β-catenin signaling pathway (Qin *et al.*, 2010). Wnt/β-catenin signaling has an important role in communication between epithelial and stromal cells in endometrium (Tulac *et al.*, 2003), and may have a role in endometriosis-related infertility, and/or its development through sex hormone homeostasis regulation (Matsuzaki *et al.*, 2010; Wang *et al.*, 2010) and fibrogenesis (Matsuzaki and Darcha, 2013). *In vitro* studies have also shown that, through targeting Wnt/β-catenin pathway, cellular mechanisms known to be involved in endometriotic lesion development, such as cell proliferation, migration and invasion of endometrial and endometriotic epithelial and stromal cells, can be inhibited (Matsuzaki and Darcha, 2013). Moreover, the Wnt/β-catenin pathway is involved in development, tissue self-renewal and in various cancers and other disease such as type II diabetes and osteoporosis (Clevers, 2006; Klaus and Birchmeier, 2008; Clevers and Nusse, 2012). *MiRNA_148a* has been shown to regulate adipogenesis through modulation of Wnt/β-catenin signaling pathway (Qin *et al.*, 2010), emphasizing the potentially important role of this pathway for both endometriosis and fat distribution. More distant candidate genes for this 7p15.2 endometriosis association signal include *HOXA10* (Homeobox A10) and *HOXA11* (Homeobox A11) which are ∼1.35 Mb downstream. *HOXA10* and *HOXA11* are members of the homeobox A family of transcription factors that play a role in uterine development (Taylor *et al.*, 1999; Wu *et al.*, 2005). It is possible that the 7p15.2 signal influences the regulation of interactive expression of a number of these genomic loci; functional studies targeting this region will need to be conducted to further elucidate its role in endometriosis pathogenesis.

Two further inter-genic loci did not reach genome-wide significance of association with endometriosis on meta-analysis, even when allowing for heterogeneic effects across studies. Rs6734792 on 2q23.3, *P* = 2.2 × 10^−6^ with all-stage endometriosis, is located 280 kb upstream of *RND3* (Rho Family GTPase 3). The signal showed similar strength of association for all-stage and Stage III/IV endometriosis, implying a potential role in the aetiology of both minimal/mild and moderate/severe disease. *RND3* encodes a member of a subgroup of Rho family of small GTP-binding proteins. Rho GTPases are key regulators of the actin cytoskeleton and stress fibre formation. In addition, *RND3* is also involved in the regulation of cell-cycle progression, cell transformation (Chardin, 2006) and cell migration (Guasch *et al.*, 1998). The third inter-genic signal, rs4141819 on 2p14 (borderline significant with *P* = 9.2 × 10^−8^ in Stage III/IV-enriched analysis) is located 227 kb away from *ETAA1* (Ewing's tumor-associated antigen 1) that encodes a tumour-specific cell surface antigen in Ewing family of tumours (EFTs). EFTs is a group of cancers that form in bone or soft tissue that share common features as they develop from the same type of stem cell in the body (Borowski *et al.*, 2006). Rs4141819 is located in an intronic region of a long non-coding RNA (lncRNA) AC007422.1, which is 118 kb long. Non-coding RNAs are functional RNA molecules that are not translated into proteins but have a regulatory role in gene expression. The biological role of this particular lncRNA is not yet known.

The remaining seven variants in six loci are located in or near (within 50 kb) genes (Fig. 4): *WNT4*, *GREB1*, *FN1*, *ID4*, *CDKN2B-AS1* and *VEZT*. These variants are likely to be involved in the ‘*cis*’-regulation of expression of neighbouring genes and/or transcripts. The current knowledge on the biological function of the implicated genes, which vary from *WNT4* and *FN1* in developmental pathways to functions of *VEZT*, *GREB1*, *ID4*, *NFE2L3*, *FN1*, *ETAA1* and *CDKN2B-AS1* in carcinogenesis, is described below.

#### WNT4 (Wingless-type MMTV integration site family member 4)

Rs7521902 is located 21 kb up/downstream of *WNT4* (Fig. 4). *WNT4* is a protein-coding gene that is vital for development of the female reproductive organs. In knockout mice, the loss of *WNT4* leads to complete absence of the Müllerian duct and its derivatives (Vainio *et al.*, 1999). A previous study investigated the expression of genes playing decisive roles during the female reproductive tract development including *WNT4* in peritoneal tissue from endometriosis cases and controls. They showed that *WNT* genes are expressed in normal peritoneum in addition to endometrium, suggesting that endometriosis can arise through metaplasia and can in the process make use of the developmental steps involved in the embryonic development of the female reproductive tract (Gaetje *et al.*, 2007). As mentioned before, *Wn*t signaling is important for epithelial–stromal cell communication in the endometrium, and is likely to be important for endometrial development, differentiation and embryonic implantation (Tulac *et al.*, 2003). Furthermore, the endometriosis variant rs7521902 has also been genome-wide significantly associated with bone mineral density (Estrada *et al.*, 2012), highlighting this variant as a potential pleiotropic locus (Fig. 4).

#### GREB1 (Growth regulation by estrogen in breast cancer 1)

Rs13394619 is located in an intronic region between exon 9 and exon 10 in *GREB1. GREB1* encodes for an early response gene in the estrogen regulation pathway that is involved in hormone-dependent breast cancer cell growth (Rae *et al.*, 2005). Furthermore, Pellegrini *et al.* (2012) showed increased expression of *GREB1* in peritoneal eutopic endometriotic lesions compared with eutopic endometrium, implicating its transcription in estrogen-dependent growth in endometriosis. The underlying biological mechanism by which *GREB1* plays a role in hormone-responsive tissues especially estrogen-stimulated cell proliferation in endometriosis remains to be elucidated. The *GREB1* region harbours many other SNPs reported to be genome-wide significantly associated with other traits and conditions, with *GREB1* in particular associated with obesity-related traits (Supplementary data, Table SI). However, none of these SNPs are in LD with endometriosis SNP rs13394619.

#### FN1 (Fibronectin 1)

Rs1250248 is located in an intronic region between exon 10 and exon 11 in *FN1* (Fig. 4). *FN1* is involved in cell adhesion and migration processes including embryogenesis, wound healing, blood coagulation, host defense and metastasis (Pankov and Yamada, 2002). Recently, it has been shown that *SOX2*, a gene encoding a transcription factor that targets *FN1*, is a key gene regulating cell migration in ovarian cancer (Lou *et al.*, 2013). Furthermore, an *in vitro* study has shown that *FN1* modulates CpG motif-dependent cytokine production in macrophages, supressing the immune resposiveness through *TLR9* pathway (Yoshida *et al.*, 2012). The *FN1* region has been associated with many other traits in GWASs (Supplementary data, Table SI); however, none of these SNPs are in LD with endometriosis SNP rs1250248.

#### ID4 (Inhibitor of DNA binding 4)

Rs7739264 is located in an intronic region of lncRNA *RP1–167F1.2* (794 bp), for which the biological function remains to be discovered. It is located 52 kb downstream of *ID4* (Fig. 4). *ID4* is an ovarian oncogene that is over-expressed in most primary ovarian cancers but not in normal ovary, fallopian tube and other tissues. Furthermore, it has been implicated, through methylation-related regulatory pathways, in breast carcinogenesis (Verschuur-Maes *et al.*, 2012) and is overexpressed in most ovarian, endometrial and breast cancer cell lines (Ren *et al.*, 2012). Potential mechanisms by which *ID4* induces transformation include, through regulation of *HOXA9* and *CDKN1A* (cyclin-dependent kinase inhibitor 1A), transcriptional programmes to disrupt the normal regulation of cell proliferation and differentiation (Ren *et al.*, 2012). *HOXA* genes have been shown to play essential roles in specifying regional differentiation of the Müllerian duct into oviduct, uterus, cervix and vagina (Kobayashi and Behringer, 2003). The region around endometriosis SNP rs7739264 contains a large number of reported SNPs associated with different traits and conditions in GWASs (Supplementary data, Table SI), none of which are in LD with rs7739264.

#### CDKN2B-AS1 (Cyclin-dependent kinase inhibitor 2B antisense RNA)

Rs1537377 is located 48 kb upstream of *CDKN2B-AS1*, while rs1333049 is located in the 3′ UTR region of the gene and rs10965235, that is monomorphic is in the European populations, is in the intron between exons 16 and 17 of the gene (Fig. 4). The *CDKN2B-AS1* locus encodes for cyclin-dependent kinase inhibitor 2B antisense RNA. In the same LD block with this gene are *CDKN2A (P16*), *CDKN2B (P15*) and *ARF (P14)*, which are all recognized tumour suppressor genes. *CDKN2B-AS1* has been shown to be involved in the regulation of *CDKN2B*, *CDKN2A* and *ARF* expression (Pasmant *et al.*, 2007; Jarinova *et al.*, 2009; Liu *et al.*, 2009). Inactivation of *CDKN2A*, through loss of heterozygosity or hypermethylation of its promoter, has been reported in endometriosis and endometrial cancer (Goumenou *et al.*, 2000; Martini *et al.*, 2002; Guida *et al.*, 2009). SNPs in or near the *CDKN2B-AS1* locus have been associated with many other traits and disease (Supplementary data, Table SI), with a number in LD (*r*^2^ > 0.2) with the endometriosis SNPs (Fig. 4), including rs1063192 and rs2157719 with glaucoma (Osman *et al.*, 2012; Wiggs *et al.*, 2012), rs4977756 with glioma (Rajaraman *et al.*, 2012), rs10757269 with ankle-brachial index (Murabito *et al.*, 2012), rs6475606 and rs10757272 with intracranial aneurysm (Foroud *et al.*, 2012; Low *et al.*, 2012), rs1537370 with coronary artery calcification (van Setten *et al.*, 2013) and rs7865618, rs10757274, rs1333042 and rs944797 with coronary heart disease (Wild *et al.*, 2011; Lu *et al.*, 2012; Takeuchi *et al.*, 2012; Lee *et al.*, 2013); because of these diverse associations, its function is an area of research for many investigators.

#### VEZT (Vezatin)

Rs10859871 is located 17 kb upstream of *VEZT* (Fig. 4). The locus was the second signal that showed similar strength of association for Stage III/IV disease [OR = 1.19 (95% CI: 1.11–1.27), *P* = 6.8 × 10^−7^] versus all endometriosis [OR = 1.18 (95% CI: 1.13–1.22, *P* = 4.8 × 10^−15^]. *VEZT* encodes an adherens junction transmembrane protein. Vezatin expression has been shown to be down-regulated in gastric cancer patients through methylation of its promoter (Guo *et al.*, 2011; Miao *et al.*, 2013). It is a putative tumour suppressor gene, targeting cell migration and invasion genes, growth genes, cellular adhesion genes and a functionally validated cell cycle progression gene called *TCF19* (transcription factor 19) (Miao *et al.*, 2013). *TCF19* was found to be associated with lymphocyte count, mean cell hemoglobin, white blood cell count, hematocrit count and eosinophil count (Ferreira *et al.*, 2009) hinting at a potential role of *TCF19* regulation through *VEZT* in maintaining immunological balance. In the Japanese population, Rs10859871 is in LD with rs12310399 (*r*^2^ = 0.57; Fig. 4, Supplementary data, Table SI) located 121 kb downstream of *VEZT*, a variant that has been associated with adverse response to chemotherapy (Low *et al.*, 2013) (Fig. 4).

## Conclusions and future directions

Our meta-analysis demonstrated directionally consistent, genome-wide association of SNPs in six genetic loci with endometriosis across European ancestry populations in Australia, Belgium, Italy, the UK, the USA, as well as Japanese ancestry populations: rs12700667 on 7p15.2, rs7521902 near *WNT4*, rs10859871 near *VEZT*, rs1537377 near *CDKN2B-AS1*, rs7739264 near *ID4* and rs13394619 in *GREB1*. Five of the six loci showed stronger effect sizes of association with Stage III/IV disease, with the exception of *VEZT*. Of the remaining three loci, two (*FN1* and inter-genic 2p14) were borderline genome wide significant for association in Stage III/IV-only or Stage III/IV-enriched analyses, respectively. Except for inter-genic rs4141819 and rs6734792 on chromosome 2, none of the other SNPs/loci showed significant evidence of heterogeneity across datasets (*P* > 0.005) showing that the significantly associated risk variants of endometriosis are pertinent to all studied populations.

It was notable that of the 11 SNPs included in the meta-analysis, 5 were in LD (*r*^2^ > 0.2) with an SNP that has also robustly been associated with different diseases and traits in other GWASs: in or near WNT4 with bone mineral density; in CDKN2B-AS1 with glaucoma, glioma, ankle-brachial index, intracranial aneurysm, coronary artery calcification and coronary heart disease; in VEZT with adverse response to chemotherapy and in 7p15.2 with fat distribution. This information is important, as the investigation of other established disease variants in genomic regions associated with endometriosis can aid in revealing the potentially biological mechanisms through which the variant may act upon endometriosis pathogenesis, and can lead to new investigations of joint aetiology and co-morbidity.

### Detailed and standardized disease phenotype

Our results showing stronger associations with Stage III/IV endometriosis emphasize the importance of detailed sub-phenotype collection to allow analyses for the identification of further variants associated with sub-types of endometriosis. Currently, datasets with detailed surgical and clinical sub-phenotype information are not available on a large scale, but such information collected in a standardized manner is required to enable future genomic research. The global WERF Endometriosis Biobanking and Phenome Harmonisation Project (EPHect), currently involving 32 clinical and basic endometriosis research centres and 3 industrial collaborators, is developing freely available data collection tools to enable standardized data collection, and thus foster future collaborative analyses across endometriosis research centres (http://www.endometriosisfoundation.org/ephect).

### Larger sample sizes

Although this may be surprising, the sample sizes of the endometriosis GWASs to date are at the lower end of GWASs in other complex disease fields, and an increased sample size for genome-wide meta-analysis of GWAS studies is predicted to increase the number of genome-wide significant loci (Visscher *et al.*, 2012). For instance, recent meta-GWAS analyses of type 2 diabetes have included close to 100 000 cases, and have identified around 65 genome-wide significant variants associated with disease (Morris *et al.*, 2012); the count of genome-wide significant loci associated with inflammatory bowel disease, involving up to 37 000 cases and controls, is as high as 163 (Jostins *et al.*, 2012). This further emphasizes the need for collaborations between centres, which collect standardized characteristics of the disease as well as detailed symptoms, to increase endometriosis case numbers and allow further GWAS datasets to be generated. Undoubtedly, larger GWASs and meta-analyses in different populations will allow the detection of additional common causal variants of modest effects on endometriosis risk.

### Better coverage of genomic variation

GWASs are limited with regard to their ability to detect only the effect of common variants (MAF > 0.05). It is likely that some of the unexplained genetic variation may be due to rarer variants (MAF < 0.05), either single sited or structural, that are not captured by current GWA genotyping arrays (Visscher *et al.*, 2012). As mentioned before, family-based linkage studies of endometriosis have successfully identified two linkage regions that are likely to harbour rare causal variants, on chromosome 10q26 (Treloar *et al.*, 2005) and on chromosome 7p13-15 (Zondervan *et al.*, 2007). Systematic resequencing studies of these regions are required to identify the rare variants involved.

### Functional studies

To understand the roles of the identified genetic variants in endometriosis, functional studies are needed tissues relevant to endometriosis, such as eutopic and ectopic endometrium. Functional studies aim to understand how changes in the DNA level of variation translate to the (regulation of) RNA transcript levels, protein levels and metabolite levels. These studies are crucial in revealing the biological mechanisms by which the genetic variations detected are causally related to the disease end-points. To allow replication of findings between studies, and collaborative analyses, centres collecting tissues for the purpose of endometriosis research need to use similar, standard-operating protocols (SOPs) for collection, processing and storage of samples. In addition to guidelines and standards for data collection, the EPHect also provides freely available consensus SOPs for biological sample collection that will allow large-scale collaborative functional studies contributing to biomarker and drug target discovery research.

## Supplementary data

Supplementary data are available at http://humupd.oxfordjournals.org/.

## Authors' roles

N.R., D.R.N., A.P.M, S.A.M, G.W.M., K.T.Z.: additional data generation; N.R., A.P.M., K.T.Z.: meta-analysis; N.R., D.R.N., A.P.M, S.A.M., G.W.M., K.T.Z.: manuscript preparation.

## Funding

D.R.N. is supported by the NHMRC Fellowship (339462 and 613674) and the ARC Future Fellowship (FT0991022) schemes. A.P.M. is supported by a Wellcome Trust Senior Research Fellowship. G.W.M. is supported by the NHMRC Fellowships Scheme (339446, 619667). K.T.Z. is supported by a Wellcome Trust Research Career Development Fellowship (WT085235/Z/08/Z). Funding to pay for the Open Access publication charges for this article was provided by the Wellcome Trust.

## Conflict of interest

There are no conflict of interests to declare.

## Supplementary Material

Supplementary Data
