# Supplementary material for: Genetic variants underlying risk of endometriosis: insights from meta-analysis of eight genome-wide association and replication datasets
Source: Hum Reprod Update. 2014 Mar 27;20(5):702–16. doi: 10.1093/humupd/dmu015 (PMC4132588; doi:10.1093/humupd/dmu015)
Supplement: Supplementary Data [file supp_20_5_702__index.html]

Genetic variants underlying risk of endometriosis: insights from meta-analysis of eight genome-wide association and replication datasets — Genetic variants underlying risk of endometriosis: insights from meta-analysis of eight genome-wide association and replication datasets — Supplementary Data 

# Genetic variants underlying risk of endometriosis: insights from meta-analysis of eight genome-wide association and replication datasets

## Supplementary Data

Supplementary Data

**Files in this Data Supplement:**

- Supplementary Table 1 - xlsx file
